# Thrombin induces IL-8/CXCL8 expression by DCLK1-dependent RhoA and YAP activation in human lung epithelial cells

**DOI:** 10.1186/s12929-022-00877-0

**Published:** 2022-11-11

**Authors:** Fara Silvia Yuliani, Jing-Yun Chen, Wen-Hao Cheng, Heng-Ching Wen, Bing-Chang Chen, Chien-Huang Lin

**Affiliations:** 1 International Graduate Program in Medicine, College of Medicine, Taipei Medical University, Taipei, Taiwan; 2 Department of Pharmacology and Therapy, Faculty of Medicine, Public Health, and Nursing, Universitas Gadjah Mada, Yogyakarta, Indonesia; 3 Graduate Institute of Medical Sciences, College of Medicine, Taipei Medical University, Taipei, Taiwan; 4 School of Respiratory Therapy College of Medicine, Taipei Medical University, Taipei, Taiwan; 5 Respiratory Therapy, Division of Pulmonary Medicine, Department of Internal Medicine, Wan Fang Hospital, Taipei Medical University, Taipei, Taiwan

**Keywords:** DCLK1, RhoA, YAP, Severe asthma, Lung epithelial cells

## Abstract

**Background:**

Doublecortin-like kinase 1 (DCLK1) has been recognized as a marker of cancer stem cell in several malignancies. Thrombin is crucial in asthma severity as it can promote IL-8/CXCL8 production in lung epithelial cells, which is a potent chemoattractant for neutrophils. However, the pathologic role of DCLK1 in asthma and its involvement in thrombin-stimulated IL-8/CXCL8 expression remain unknown.

**Methods:**

IL-8/CXCL8, thrombin, and DCLK1 expression were observed in the lung tissues of severe asthma patients and ovalbumin (OVA)-induced asthmatic mice model. A549 and BEAS-2B cells were either pretreated with inhibitors or small interfering RNAs (siRNAs) before being treated with thrombin. IL-8/CXCL8 expression and the molecules involved in signaling pathway were performed using ELISA, luciferase activity assay, Western blot, or ChIP assay.

**Results:**

IL-8/CXCL8, thrombin, and DCLK1 were overexpressed in the lung tissues of severe asthma patients and ovalbumin (OVA)-induced asthmatic mice model. Our in vitro study found that DCLK siRNA or LRKK2-IN-1 (DCLK1 inhibitor) attenuated IL-8/CXCL8 release after thrombin induction in A549 and BEAS-2B cells. Thrombin activated DCLK1, RhoA, and YAP in a time-dependent manner, in which DCLK1 siRNA inhibited RhoA and YAP activation. YAP was dephosphorylated on the Ser127 site after thrombin stimulation, resulting in YAP translocation to the nucleus from the cytosol. DCLK1, RhoA and YAP activation following thrombin stimulation were inhibited by U0126 (ERK inhibitor). Moreover, DCLK1 and YAP siRNA inhibited κB-luciferase activity. Thrombin stimulated the recruitment of YAP and p65 to the NF-κB site of the IL-8/CXCL8 promoter and was inhibited by DCLK1 siRNA.

**Conclusions:**

Thrombin activates the DCLK1/RhoA signaling pathway, which promotes YAP activation and translocation to the nucleus from the cytosol, resulting in YAP/p65 formation, and binding to the NF-κB site, which enhances IL-8/CXCL8 expression. DCLK1 might be essential in thrombin-stimulated IL-8/CXCL8 expression in asthmatic lungs and indicates a potential therapeutic strategy for severe asthma treatment.

## Background

Severe asthma is found in less than 10% of all patients with asthma; however, it has the greatest impact on morbidity, quality of life, and financial burden among the overall asthmatic population [1, 2, 3]. Asthma is typically easy to manage; however, even when maximal inhaled therapy (inhaled corticosteroids and long-acting 2 agonists) is used, certain individuals with severe asthma are poorly managed [4]. Among these are neutrophilic asthma patients. This type of asthma exhibits a high neutrophil count; severe, persistent, and frequent exacerbations; and fixed airway obstruction [5]. Therefore, we need to explore the pathogenic mechanism of severe asthma, particularly neutrophilic asthma, and target proteins that can be used as future drug candidates.

Thrombin is a versatile (multipurpose) serine protease that is essential in platelet activation and blood coagulation [6]. Apart from its main role in homeostasis, thrombin catalyzes the cleavage of fibrinogen to fibrin in order to form a blood clot following tissue injury [7, 8]. When air capillaries are damaged, thrombin is released, which then binds to the protease-activated receptor (PAR)1, PAR3, and PAR4 on the cell membrane of lung epithelial cells, secreting many inflammatory substances, for example IL-6, PGE_2_, and IL-8/CXCL8, which are involved in chronic airway inflammation [8, 9]. A large amount of thrombin was shown in the sputum and bronchoalveolar of asthma patients after allergen exposure [10]. Macrophage inflammatory protein 2 (MIP-2), also known as IL-8/CXCL8 in mice, increases significantly in bronchoalveolar lavage fluid after thrombin stimulation via the airway [9]. We then demonstrated in an in vitro study that thrombin-stimulated IL-8/CXCL8 expression involves NF-κB activation in lung epithelial cells [11]. These studies show that thrombin is crucial in the inflammation of the respiratory tract by inducing IL-8/CXCL8.

IL-8/CXCL8 is a major chemokine that promotes neutrophils recruitment to the inflamed area. Infiltrated neutrophils can worsen the inflammatory response by releasing some mediators, which causes damage to the respiratory tract tissue, mucus hypersecretion, and insensitivity to steroids [12, 13, 14]. This persistent condition can eventually result in fibrosis of the respiratory tract and a decrease in respiratory function [14, 15, 16]. Thus, IL-8/CXCL8 has an important crucial pathological role in airway inflammation in severe asthma.

Doublecortin-like kinase 1 (DCLK1) is a serine/threonine protein kinase, a member of the microtubule-associated protein family, and regulates inflammatory genes [17]. It was first discovered to play a role in neuronal migration and neurogenesis [18, 19]. When DCLK1 is phosphorylated at Ser30, it increases its activity. Previous study has shown that DCLK1 Ser30 is phosphorylated by extracellular signal-regulated kinase (ERK), that can improve the performance of endocrine melanocyte pro-opiomelanocortin [20]. Moreover, DCLK1 is a cancer stem cells marker in intestinal tumor cells and contributes to breast cancer cell metastases [21, 22]. The activation of S100A9/NF-κB pathway in hepatitis C virus via DCLK1 induces cirrhosis, and DCLK1 overexpression is positively correlated with the expression of inflammatory genes [23]. Prior study has shown that severe asthma patients exhibit increased IL-17 levels in their airways [24]. Moreover, IL-17 drives the upregulation of DCLK1 expression in a bleomycin-induced lung fibrosis mice model and pancreatic intraepithelial neoplasia cells [25, 26]. Based on the accumulated evidence, DCLK1 overexpression in different cells plays an important pathological role in various diseases, but whether DCLK1 is involved in severe asthma remains unknown.

RhoA is a small GTPase that can be activated by PAR and is important in the activation of lung inflammatory genes [27]. A prior study found that RhoA mediates NF-κB activation and IL-8/CXCL8 production induced by lipopolysaccharide (LPS) in respiratory tract epithelial cells [28]. In addition, in macrophages, the activation of PI3K pathway can phosphorylate the ezrin/radixin/moesin complex to promote RhoA translocation to the cell membrane, increasing RhoA activity [29]. Moreover, RhoA can be stimulated by thrombin to activate yes-associated protein 1 (YAP) through PAR1 [30]. YAP is a transcriptional co-activator that is also involved in asthma and cancer pathogenesis [31, 32]. A large amount of YAP can be used as a biomarker to assess asthma severity [33]. When YAP is activated following dephosphorylation of its Ser127 site, which is mediated by RhoA activation, it enters the nucleus from the cytosol and binds with transcription factors such as NF-κB p65 and TEAD to increase gene transcription [13, 34]. However, whether DCLK1 induces the activation of RhoA and YAP to regulate thrombin-stimulated IL-8/CXCL8 expression in lung epithelial cells remains unclear.

In this study, IL-8/CXCL8, thrombin, and DCLK1 are found to be overexpressed in the lung tissues of severe asthma patient and OVA-challenged asthmatic mice model. Thrombin stimulates DCLK1 and RhoA, which causes YAP to be activated and translocated to the nucleus from the cytosol, where it interacts with NF-κB p65 and binds to IL-8/CXCL8 promoter to induce its production in human lung epithelial cells.

### Methods

## Materials

The human lung/bronchial epithelial cell line BEAS-2B (CRL-9609®) and human lung epithelial cell line A549 (CCL-185®) were procured from the American Type Culture Collection (ATCC) (Manassas, VA, USA). Control siRNA (scrambled), U0126, α-tubulin antibody (T9026), ovalbumin (OVA), and thrombin were obtained from Sigma-Aldrich (Saint Louis, MO, USA). Aluminum hydroxide (Alum) was procured from Thermo Fisher Scientific (Waltham, MA, USA). Anti-RhoA (sc-418), protein A/G beads, anti-rabbit IgG (sc-2004), anti-mouse IgG (sc-2314) were obtained from Santa Cruz Biotechnology (CA, USA). The antibodies for IL-8/CXCL8 (bs-0780R) and MIP-2 (PA547015) were obtained from Bioss (Woburn, MA, USA) and Invitrogen (Waltham, MA, USA), respectively. The specific antibodies for DCLK1 (ab31704), YAP1 (ab52771), YAP phospho Ser127 (ab76252), thrombin (ab92621), and E-cadherin (ab40772) were procured from Abcam (Cambridge, UK). Anti-DCLK1 phospho Ser30 was procured from Leadgene (Taiwan). Bronchial epithelial growth medium (BEGM) was attained from Lonza (Verviers, Belgium). Dulbecco’s modified Eagle’s medium/Nutrient Mixture F-12 (DMEM/F-12), fetal bovine serum (FBS), Lipofectamine-3000 were procured from Life Technologies (Carlsbad, CA, USA). An immunohistochemistry assay was performed using a Novolink™ Max Polymer Detection System (Leica Microsystems, Wetzlar, Germany). An enzyme-linked immunosorbent assay (ELISA) for IL-8/CXCL8 measurement was performed using R&D Systems. A pBK-CMV-*Lac Z* (*LacZ*) was kindly supplied by Dr. W.W. Lin (National Taiwan University, Taipei, Taiwan). RhoA activation assay kit was obtained from Millipore (Billerica, MA, USA). An assay kit of chromatin immunoprecipitation (ChIP) was attained from Upstate Biotechnology Millipore (Lake Placid, NY, USA). The pcDNA and IL-8/CXCL8 wildtype (-133) luciferase (IL-8/CXCL8 WT-Luc) were generously given by Dr. M.C. Chen (Taipei Medical University, Taipei, Taiwan) and Dr. Naofumi Mukaida (Kanazawa University, Kanazawa, Japan), respectively.

### Human subject

Healthy subjects (normal) with normal lung function, asthma patients with normal lung function (mild asthma) who had predicted forced expiratory volume in 1 s (FEV1) of > 80%, and severe asthma patients with lung function impairment (FEV1 < 60%) were enrolled. Endobronchial biopsy was performed to collect the participants' lung tissues, which were then used for immunohistochemistry (IHC) and immunofluorescence (IF) staining. All participants provided informed consent. All studies involving human subjects were approved by Taipei Medical University-Joint Institutional Review Board (TMU-JIRB No. N201702033).

### OVA‐induced asthmatic animal model

An OVA-induced asthmatic animal model was developed using 6–8-weeks-old C57BL/6 mice (BioLASCO, Taipei, Taiwan) by administering 10 µg/0.1 ml OVA intraperitoneally on days 1, 8, and 15. From week 4 to week 11, the mice was exposed to phosphate-buffered saline (PBS) alone or aerosolized OVA (5% wt/vol in PBS) twice a week for 8 weeks. The mice were sacrificed after week 12^th^, and the lung tissues were examined for Western blot, IHC, and IF staining. All protocols for animal experiments were approved by the Animal Ethics Committee of Taipei Medical University (No. LAC-2019-0042).

### Cell culture and transfection

A549 and BEAS-2B cells were cultured in DMEM/F-12 and BEGM, respectively, in a 5% CO_2_ incubator at 37 °C. Once achieving confluence, the cells were seeded onto 24- and 12-well plates for ELISA and luciferase assay, respectively. Moreover, 6 cm dishes for Western blot, and 10 cm dishes were used for co-immunoprecipitation and ChIP assay. For transient transfection of siRNA into A549 and BEAS-2B cells, the transfection reagent/siRNA mixture was incubated at room temperature for 10 min and then added dropwise to the cell culture medium; then incubated in a humidified 37 °C incubator for 48 h. The following sequences were used for DCLK1 siRNAs (#1: sense, 5ʹ-GAUCGAUACUUCAAAGGGA-3ʹ; anti-sense, 5ʹ-UCCCUUUGAAGUAUCGAUC-3ʹ, #2: sense, 5ʹ-CAAAGGAGCUCAUUACCAU-3ʹ; anti-sense, 5ʹ-AUGGUAAUGAGCUCCUUUG-3ʹ) and YAP siRNAs (#1: sense, 5ʹ-CACCUAUCACUCUCGAGAU-3ʹ; anti-sense, 5ʹ-AUCUCGAGAGUGAUAGGUG-3 ʹ, #2: sense, 5ʹ-GCUCAUUCCUCUCCAGCUU-3ʹ; anti-sense, 5ʹ-AAGCUGGAGAGGAAUGAGC-3ʹ). For DCLK1 overexpression, A549 cells were transfected with DCLK1 wild type (WT) or DCLK1 kinase dead (KD) or empty vector (pcDNA) by Lipofectamine.

### Immunohistochemistry staining

The paraffin-embedded human lung tissues were deparaffinized, rehydrated, and antigen retrieval-treated before performing IHC staining. Peroxidase blocking was used to neutralize endogenous peroxidase in the tissue. After that, the tissue was blocked using a blocking solution and treated for 40 min with antibodies against thrombin, IL-8/CXCL8, DCLK1, phospho-DCLK1, RhoA, and phospho-YAP followed by 30 min with secondary antibodies. Hematoxylin and DAB solutions were used to stain the tissues.

### Immunofluorescence staining

The paraffin-embedded lung tissue sections of mice were processed for antigen retrieval. The tissue was blocked using a blocking solution, treated with a DCLK1 specific antibody, and then immunostained with conjugated secondary antibodies (Alexa Fluor 488). For DCLK1, IL-8/CXCL8, and thrombin triple positive staining, the slides were blocked using blocking solution, then incubated with antibodies specific to DCLK1- Alexa flour 488 conjugated antibody for 1 h. Afterward, the slides were incubated with IL-8/CXCL8 antibody for 1 h, then, an incubation with goat anti-rabbit IgG (H&L) and donkey anti-goat IgG (H&L) Alexa fluor 555 for 1 h. The slides were incubated with antibodies specific to thrombin overnight (4℃) following by a goat anti-rabbit IgG (H&L) Alexa fluor 647 for another 1 h. Nuclear were counterstained by DAPI with mounting medium. The mean fluorescence intensity (MFI) of protein expression was measured and divided by the total number of DAPI positive cells to determine the average protein intensity of each cells using ImageJ software (National Institute of Health, Bethesda, MD, USA).

For immunocytochemistry (ICC) staining, A549 cells were seeded onto slides and preserved with paraformaldehyde 4% after 30 min of thrombin (10 U/ml) stimulation. Before being washed in PBS, the cells were permeabilized in PBS containing 0.5% Triton X-100. Afterwards, the cells were blocked using a blocking buffer and treated with anti-YAP antibodies overnight at 4 °C. The cells were incubated with conjugated secondary antibody (Alexa Fluor 555) for 1 h. DAPI was used for counter-staining, and the glass slide was fixed with mounting media. A fluorescent microscope was used to examine the slides. Positive cells for YAP in the nucleus were then counted and divided by the total number of nuclei (DAPI).

### Luciferase activity assay

A549 cells (5 × 10^4^ cells/well) were grown overnight and transfected with 0.2 μg of IL-8/CXCL8-Luciferase (IL-8 Luc), 0.5 μg of κB-Luc, and *LacZ* with Lipofectamine 3000 for 24 h. The cells were then stimulated for 16 h with thrombin (10 U/ml) before being collected. The luciferase activity level was normalized to *LacZ*.

### IL-8/CXCL8 measurement

A549 or BEAS2B cells (1 × 10^5^ cells/well) in 24-well plates were incubated with 10 U/ml thrombin. The cells’ culture medium was collected and performed following the ELISA kit instructions to measure IL-8/CXCL8 release.

### RhoA activity assay

The RhoA activation assay was performed according to the manufacturer's protocol to determine the amount of activated RhoA (Rho-GTP). A549 cells in 10 cm dishes were washed with ice-cold Tris-buffered saline, lysed with ice-cold Mg^2+^ lysis/wash buffer containing leupeptin, aprotinin, phosphatase inhibitor, and sodium orthovanadate, and then agitated in 4 °C. The samples were reacted with Rhotekin RBD-agarose which binds selectively to Rho-GTP, not Rho-GDP. A primary antibody for anti-Rho was used to visualize the protein by Western blot analyses.

### Isolation of membrane fraction

A549 cells in 10 cm dishes were washed with PBS and lysed in homogenization buffer (2 mM EDTA, 5 mM EGTA, 20 mM Tris–HCl, 1 mM dithiothreitol, and 1 mM sodium orthovanadate) and stored in − 80 °C overnight. Cell lysates were then disrupted by a 3/10 mL BD™ insulin syringe and the pellets were obtained after centrifugation. The cytosolic fraction was obtained from the supernatant. Pellets were immersed with PBS, and then homogenized in the Mg^2+^ Lysis/Wash Buffer (MLB) containing protease inhibitor cocktail on ice, and centrifuged. The supernatant was collected as the membrane fraction. Proteins of membrane fractions were examined for RhoA by Western blot analyses.

### Western blot analysis

A549 cells were harvested after thrombin stimulation. SDS-PAGE was used to separate whole-cell lysates, which were then transferred to a membrane (polyvinylidene difluoride (PVDF)) and immersed in blocking solution before being incubated in primary antibodies at 4 °C overnight. Antibodies specific to DCLK1, DCLK1 phospho Ser30, RhoA-GTP, RhoA, E-cadherin, YAP, or YAP phospho Ser127 were used to visualize the proteins. The membrane was then incubated in an HRP-conjugated secondary antibody. Enhanced chemiluminescence reagents were used to identify the blots. Quantitative data were attained using ImageJ analysis software.

### Co-immunoprecipitation

A549 cells in 10 cm dishes were treated with thrombin (10 U/ml). The cells were harvested and centrifuged. Using protein A beads, the supernatants were immunoprecipitated overnight at 4 °C with antibodies specific to YAP1 or p65. The samples were examined using Western blot.

### ChIP assay

A549 cells in 10 cm dishes were treated with thrombin (10 U/ml) for 30 min. Afterwards, the cells were incubated in 10% formaldehyde for 10 min before being harvested and sonicated. Afterwards, anti-YAP, anti-p65, or rabbit anti-IgG antibodies was applied for immunoprecipitation (using protein A beads). A polymerase chain reaction (PCR) with the primer sequences: IL-8/CXCL8 promoter region (sense, 5ʹ-TCACCAAATTGTGGAGCTTCAGTAT-3ʹ; anti-sense, 5ʹ-GGCTCTTGTGCTTGTG

T-3′) was used to amplify the NF-κB response element on the IL-8/CXCL8 promoter. The PCR product was evaluated using 2% agarose gel electrophoresis.

### Quantitative PCR

A549 cells were washed with PBS, and total RNA was extracted with Nucleozol. qPCR was performed following the manufacturer's instructions. The following sequences were used for qPCR primer: DCLK1 (sense, 5′-AGCCTCATGCGGTTAATTTG-3′; anti-sense, 5′-TCTTTGAATTAGCGCCTGGT-3′), YAP (sense, 5′-CAGAACCGTTTCCCAGACTAC-3′; anti-sense, 5′-ATCAGCTCCTCTCCTTCTATGT-3′), RhoA (sense, 5′-GTACATGGAGTGTTCAG

CAAAGACC-3′; anti-sense, 5′-GGTGGGCCAGACGGGTTGGACA-3′), p65 (sense, 5′-TGTGTTCACAGACCTGGCATCCGTCGACAA-3′; anti-sense, 5′-AGCAGGAG

AAGTCCATGTCCGCAATG-3′), and GAPDH (sense, 5′-GTCTCCTCTGACTTCA

ACAGCG-3′; anti-sense, 5′-ACCACCCTGTTGCTGTAGCCAA-3′). RNA concentrations were determined using a spectrophotometer (NanoDrop® ND-1000, Thermo Scientific). RNA was reverse transcribed to cDNA using the reverse transcription kit. SYBR green Master Mix and Rotor-Gene Q PCR Detection System (Chatsworth, CA, USA) were used to perform real-time PCR. Each sample was tested three times.

### Statistical analysis

The mean standard error of the mean (SEM) is used to present data from experiments that were done at least three times independently. A one-way ANOVA was carried out, followed by a Dunnett's test analysis. An independent sample t-test was performed to compare between two groups. The results were statistically significant if *p* < 0.05.

## Results

### DCLK1 is overexpressed in lung epithelial cells of severe asthma patients and the OVA-induced mice model of asthma

**Fig. 1:**
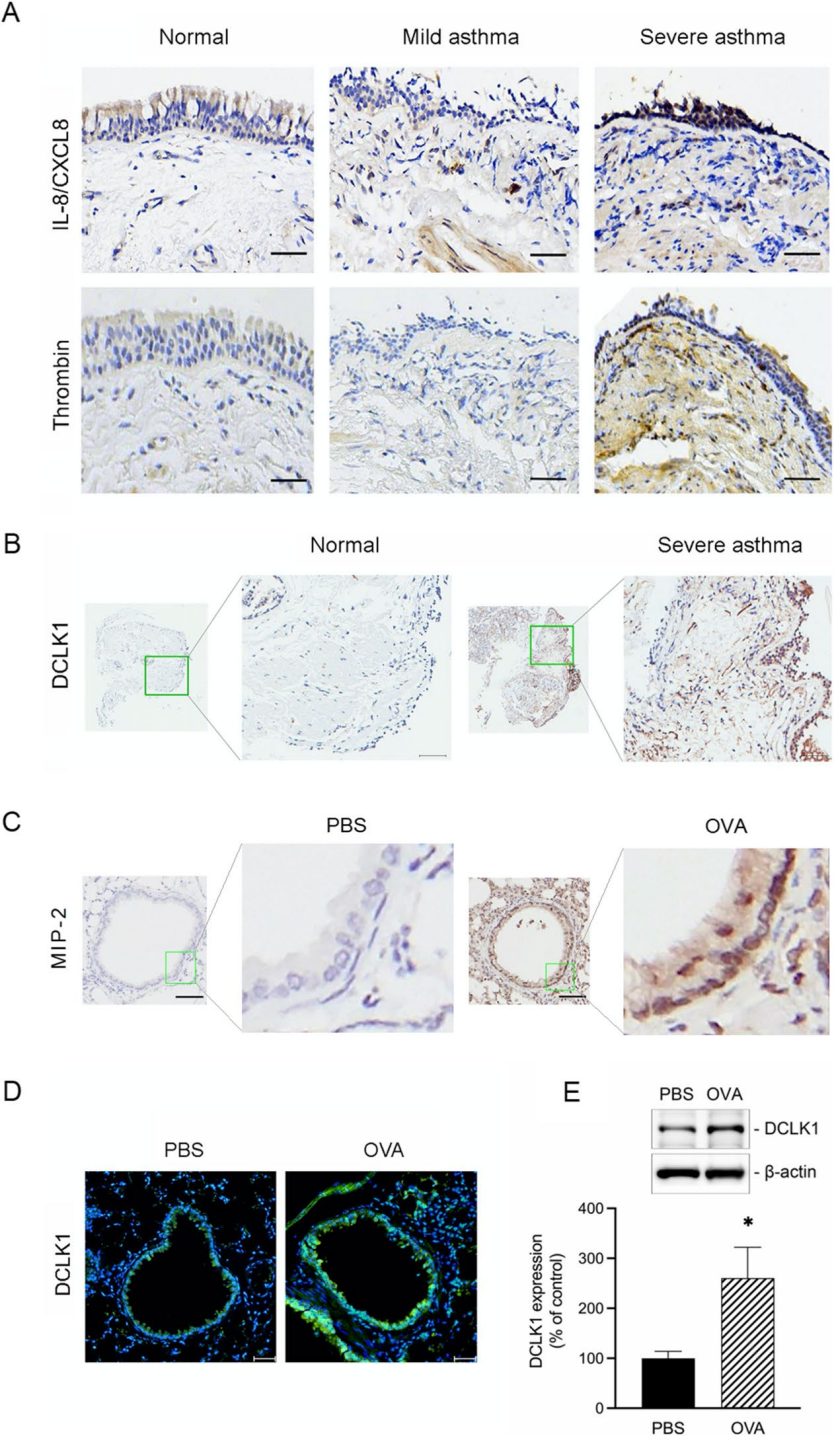

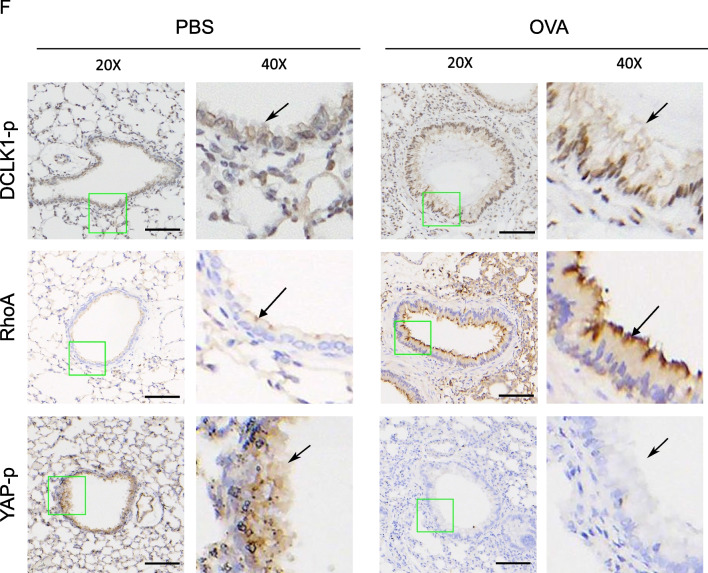
DCLK1 is overexpressed in the lungs of severe asthma patients and OVA-challenged mice. **A** Representative examples of IHC staining for IL-8/CXCL8 and thrombin in bronchial biopsies from severe asthma patients compared to normal subjects and from those with mild asthma (n = 4–6, original magnification = 20 × , bars = 100 μm). **B** IHC staining for DCLK1 in bronchial biopsies of severe asthma patients compared with normal subjects (n = 5, original magnification = ×20, bars = 50 μm). **C** Representative examples of IHC staining for MIP-2 in the lung tissues of PBS-treated and OVA-challenged mice (n = 6, original magnification = ×20, bars = 100 μm). **D** Lung tissues from PBS-treated and OVA-challenged mice were detected for the DCLK1 (green) and nuclei (blue). Merged fluorescence microphotographs were captured by immunofluorescence microscopy (n = 3, original magnification = ×20, bars = 50 μm). **E** Whole lung lysates from PBS-treated and OVA-challenged mice were immunoblotted with antibodies specific for DCLK1 and β-actin (n = 6, mean ± SEM, *p < 0.05 vs PBS group). **F** Representative examples of IHC staining for DCLK1 phosphorylation, RhoA, and YAP phosphorylation from the lung tissues of PBS-treated and OVA-challenged mice (n = 6, original magnification = ×20, bars = 100 μm)

First, we observed the bronchial tissues of the patients with different severity to express thrombin and IL-8/CXCL8. According to the literature, those molecules are increased in the asthma patients’ sputum [10]. We confirmed that the patients with severe asthma exhibited higher amounts of IL-8/CXCL8 expression in their lung tissues, especially epithelial cells, than normal subjects or mild asthma patients (Fig. 1A upper panel). Likewise, thrombin expression was overexpressed in the lung tissues and epithelial cells of severe asthma patients compared to normal subjects or mild asthma patients (Fig. 1A bottom panel). However, IL-8/CXCL8 and thrombin expression in lung tissues of patients with mild asthma was not significantly different from that in the normal subjects (Fig. 1A).

According to the literature, patients with severe asthma exhibit increased IL-17 levels in their airways [24]. Recently, it has also been found that IL-17 drives the induction of DCLK1 expression in a bleomycin-lung fibrosis mice model and pancreatic intraepithelial neoplasia cells [25, 26]. Next, we examined whether the bronchial tissues of patients with severe asthma would overexpress DCLK1. As shown in Fig. 1B, patients with severe asthma had higher DCLK1 expression in their bronchial tissues and epithelial cells than the normal subjects.

Our previous study demonstrated that MIP-2, murine homologue of IL-8/CXCL8, increases significantly in bronchoalveolar lavage fluid after thrombin stimulation via the airway in mice [9]. In this study, we confirmed that OVA-challenged mice induced MIP-2 expression in bronchial epithelial cells and subepithelial layer (Fig. 1C). Furthermore, we investigated whether OVA-challenged mice can induce DCLK1 expression in bronchial epithelial cells. We confirmed that OVA also increased the number of DCLK1-expressing bronchial epithelial cells and subepithelial layer (Fig. 1D). Similarly, OVA-stimulated mice increased DCLK1 protein by 2.6 ± 0.6 folds compared to the control group (PBS), as shown in Fig. 1E.

Afterward, we performed immunohistochemistry staining to confirm the involvement of DCLK1 phosphorylation, RhoA, and YAP dephosphorylation in OVA-challenged mice. As shown in Fig. 1F, DCLK1 activation, as indicated by DCLK1 phosphorylation on Ser30, was increased in OVA-challenged mice. Previous study has shown that overexpression of RhoA is involved in the pathogenesis of asthma [35]. It has been reported that RhoA activation is increased in airway epithelial cells of mouse model of asthma [36]. Our IHC staining showed that RhoA expression was elevated in OVA-challenged mice, indicating the involvement of RhoA in asthma pathogenesis. Prior finding has shown that the amount of YAP phosphorylation is reduced in the lung lysates of OVA-challenged mice [37]. Similarly, we confirmed that the level of YAP phosphorylation on Ser127 site was lower in lung tissues of OVA-challenged mice compared to PBS showing its activation. These results showed that DCLK1 is overexpressed in the bronchial epithelial cells of severe asthma patients or OVA-induced asthmatic mice.

### Thrombin, IL-8/CXCL8 (MIP-2) and DCLK1 are highly expressed in lung tissues of severe asthma patients or OVA-induced mice model of asthma

**Fig. 2:**
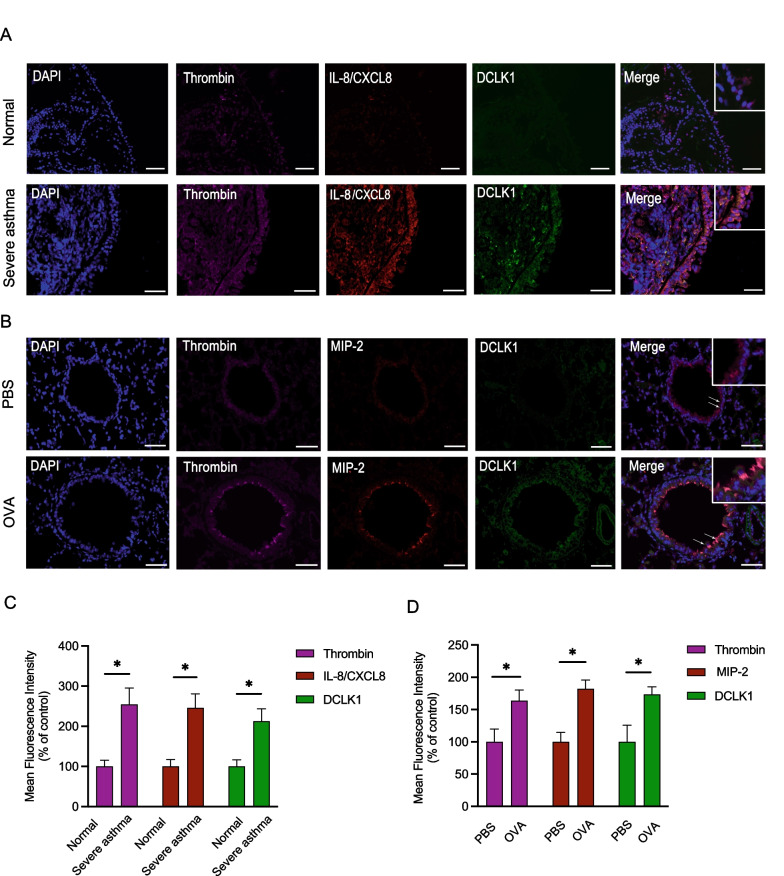
Triple immunofluorescence staining of thrombin, IL-8/CXCL8 (MIP-2), and DCLK1. **A** Representative examples of triple immunofluorescence staining for thrombin, IL-8/CXCL8, and DCLK1 in bronchial biopsies from severe asthma patients compared to normal subjects (n = 4–5, original magnification = ×20), and **B** OVA-challenged compared to PBS-treated mice (n = 5–7, original magnification = ×20). The merged image demonstrates colocalization of thrombin, IL-8/CXCL8, MIP-2, and DCLK1. The Image J software was used to measure the mean fluorescence intensity (MFI) of thrombin, IL-8/CXCL8 (MIP-2), and DCLK1 from **C** severe asthma patients compared to normal subjects (n = 4–5, mean ± SEM, *p < 0.05 vs normal) and **D** OVA-challenged compared to PBS-treated mice (n = 5–7, mean ± SEM, *p < 0.05 vs PBS)

To confirm whether thrombin, IL-8/CXCL8 (MIP-2), and DCLK1 are highly expressed in lung tissues of asthma patients and OVA-induced mice, we performed triple staining immunofluorescence. The IL-8/CXCL8 gene is lacking in mice, and it has been demonstrated that mouse MIP-2 are functional homologs of human IL-8/CXCL8 [38]. Figure 2A, B shows triple immunofluorescence staining of thrombin, IL-8/CXCL8 (MIP-2), and DCLK1 in the lung tissues of normal and severe asthma patients, as well as PBS and OVA-challenged mice. The merge images show the colocalization of thrombin, IL-8/CXCL8 (MIP-2), and DCLK1 in the lung sections of severe asthma patients or OVA-challenged mice. The quantitative analysis indicates a significant increases in the mean intensity fluorescence (MFI) of thrombin by 254.1 ± 41.1%, IL-8/CXCL8 by 245.7 ± 35.0%, and DCLK1 by 212.4 ± 31.0% in severe asthma patients compared to normal (Fig. 2C). In addition, Fig. 2D demonstrates the higher expression of thrombin by 163.8 ± 16.4%, MIP-2 by 182.2 ± 13.5%, and DCLK1 by 173.5 ± 11.8% in the lung tissues of OVA-challenged mice compared to PBS. These findings showed that thrombin, IL-8/CXCL8 (MIP-2), or DCLK1 are overexpressed and colocalized in lung tissues of severe asthma patients or OVA-induced mice model of asthma.

### DCLK1 is involved in thrombin-induced IL-8/CXCL8 release from human lung epithelial cells

**Fig. 3:**
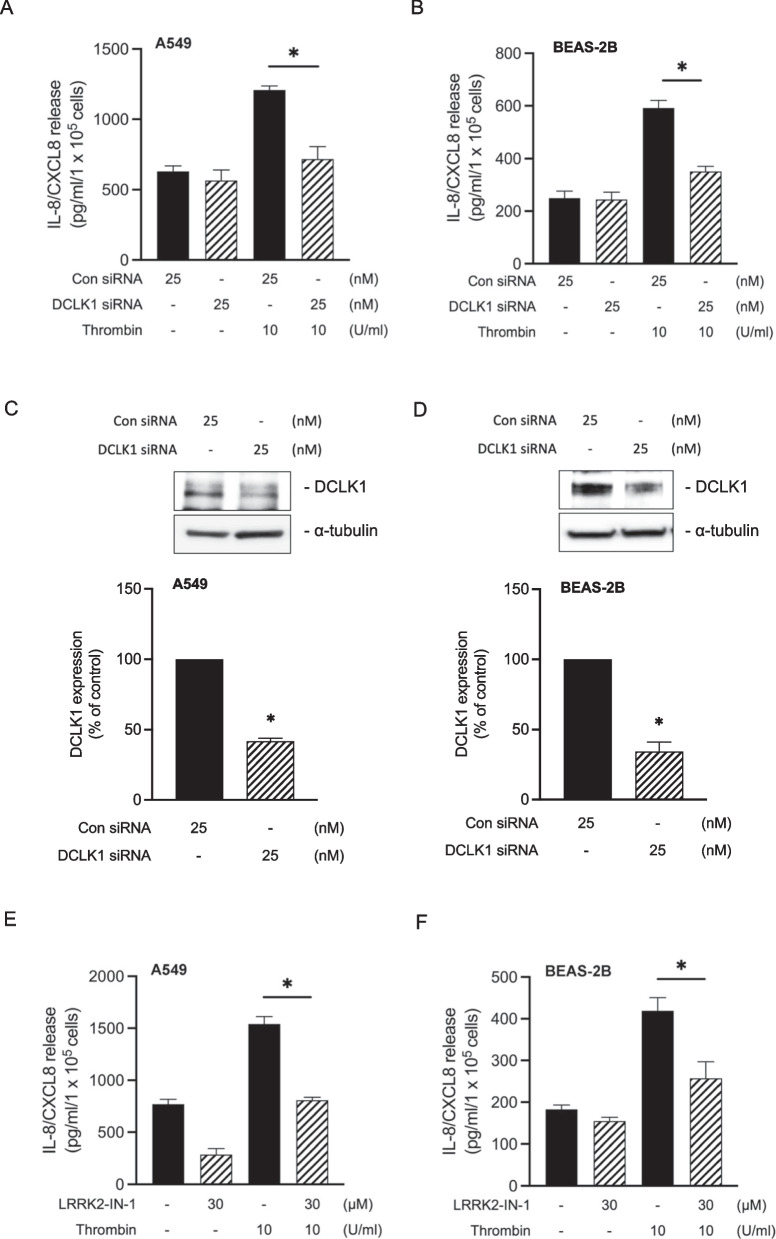
DCLK1 participates in IL-8/CXCL8 release stimulated by thrombin in A549 and BEAS-2B cells. A549 (**A**) and BEAS-2B (**B**) cells were transfected with DCLK1 siRNA or control (con siRNA) for 48 h, then treated for additional 24 h with thrombin (10 U/ml). The level of IL-8/CXCL8 release was detected using ELISA (n = 3–4, mean ± SEM, *p < 0.05 vs thrombin-stimulated group). A549 (**C**) and BEAS-2B (**D**) cells were transfected with DCLK1 siRNA or control (con siRNA) for 48 h. Immunoblotting was performed to determine the expression level of DCLK1 (n = 3, mean ± SEM, *p < 0.05 vs control). A549 (**E**) and BEAS-2B (**F**) were incubated with LRRK2-IN-1 for 30 min, and the cells were subsequently treated for additional 24 h with 10 U/ml thrombin. The level of IL-8/CXCL8 release was determined using ELISA (n = 3, mean ± SEM, *p < 0.05 vs thrombin-stimulated group)

To investigate DCLK1's role in mediating thrombin-induced IL-8/CXCL8 release, we used DCLK1 siRNA to suppress IL-8/CXCL8 release in A459 and BEAS-2B cells. We showed that DCLK1 siRNA (25 nM) suppressed thrombin-induced IL-8/CXCL8 release by 73.4 ± 15.1% (Fig. 3A). Similarly, 25 nM DCLK1 siRNA also suppressed thrombin-induced of IL-8/CXCL8 release to 68.8 ± 5.7% BEAS-2B (Fig. 3B). The transfection of DCLK1 siRNA functionally inhibited DCLK1 protein expression in A549 by 65.7 ± 6.9% (Fig. 3C) and BEAS-2B by 58.3 ± 2.2% (Fig. 3D). We subsequently used LRRK2-IN-1 (DCLK1 inhibitor) to confirm the role of DCLK1 in thrombin-stimulated IL-8/CXCL8 release. We then confirmed the involvement of DCLK1 in thrombin-stimulated IL-8/CXCL8 release, which was performed using LRRK2-IN-1. We found that 30 μM of LRRK2-IN-1 suppressed IL-8/CXCL8 release induced by thrombin in A549 to 63.2 ± 7.1% (Fig. 3E). Similarly, 30 μM LRRK2-IN-1 inhibited thrombin-induced IL-8/CXCL8 release in BEAS-2B to 56.6 ± 16.1% (Fig. 3F). Thus, DCLK1 is found to play a role in IL-8/CXCL8 release from lung epithelial cells following thrombin stimulation. Considering that DCLK1 regulation in thrombin-induced IL-8/CXCL8 release in A549 is comparable to that of the BEAS-2B response, we then used A549 for further investigations.

### DCLK1 participates in thrombin-stimulated IL-8/CXCL8 expression in A549 cells

**Fig. 4:**
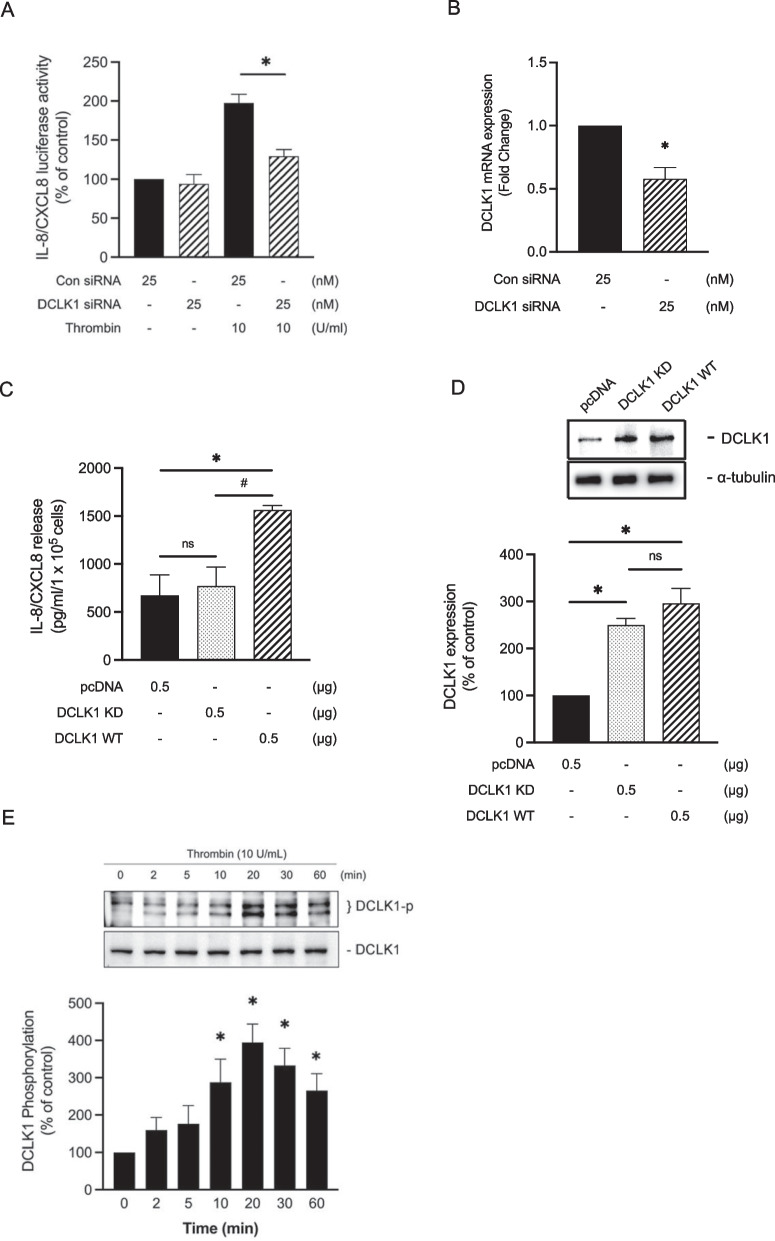
Thrombin induces IL-8/CXCL8 promoter activity via DCLK1 in A549 cells. **A** A549 cells were transfected with IL-8/CXCL8 wt-Luc (0.2 μg) and pBK-CMV-lacZ (0.1 μg), control (con siRNA), or DCLK1 siRNA for 48 h before being incubated for 16 h with thrombin (10 U/ml). Cells were collected for a luciferase activity assay (n = 6, mean ± SEM, *p < 0.05 vs thrombin-stimulated cells). **B** A549 were transfected with DCLK1 siRNA or control (con siRNA) for 48 h. We performed qPCR analysis to determine the mRNA expression of DCLK1 (n = 3, mean ± SEM, *p < 0.05 vs control group). **C** A549 cells were transfected with pcDNA or DCLK1 KD or DCLK1 WT plasmid for 48 h, and the level of IL-8/CXCL8 release was verified (n = 3, mean ± SEM, *p < 0.05 vs DCLK1 KD group, vs pcDNA group). **D** A549 cells were transfected with pcDNA or DCLK1 KD or DCLK1 WT plasmid for 48 h. Immunoblotting was performed to determine the expression level of DCLK1 (n = 3, mean ± SEM, *p < 0.05, vs pcDNA group, vs DCLK1 KD group). **E** A549 cells were induced by thrombin (10 U/ml) for 0–60 min. Levels of phospho-DCLK1 Ser30 were evaluated using Western blot (n = 5, mean ± SEM, *p < 0.05 vs non-stimulated cells)

We then used DCLK1 siRNA in A549 cells to observe whether DCLK1 mediates thrombin-induced IL-8/CXCL8 luciferase activity. DCLK1 siRNA (25 nM) significantly reduced thrombin-stimulated IL-8/CXCL8 luciferase activity by 64.2 ± 9.4%, as seen in Fig. 4A. Moreover, we confirmed using qPCR analysis that the transfection of DCLK1 siRNA in A549 cells significantly decreased the level of DCLK1 mRNA expression by a 0.4 ± 0.1 fold in (Fig. 4B). In addition, we performed A549 cells overexpressing DCLK1 by utilizing an established DCLK1 kinase-dead mutant (KD) D511N [17, 39] or DCLK1 wild-type (WT) or empty vector (pcDNA). We confirmed that A549 cells overexpressing DCLK1 WT stimulated IL-8/CXCL8 release by 230 ± 3.6% and 202 ± 3.2% compared to DCLK1 KD and pcDNA, respectively, as shown in Fig. 4C. This establishes that A549 cells are dependent on DCLK1 and its kinase activity for IL-8/CXCL8 release. As shown in Fig. 4D, DCLK1 KD and DCLK1 WT overexpression constructs showed similar levels of DCLK1 expression, and higher level compared to pcDNA by 149.8 ± 5.3% and 196.3 ± 6.8%, respectively. Next, we investigated whether thrombin would increase the phosphorylation of DCLK1. Thrombin induced the phosphorylation of DCLK1 Ser30 in A549 in time-dependent manner, with peak expression at 20 min (Fig. 4E). These findings show that DCLK1 is involved in thrombin-stimulated IL-8/CXCL8 expression in A549 cells.

### RhoA is involved in thrombin-stimulated IL-8/CXCL8 expression

**Fig. 5:**
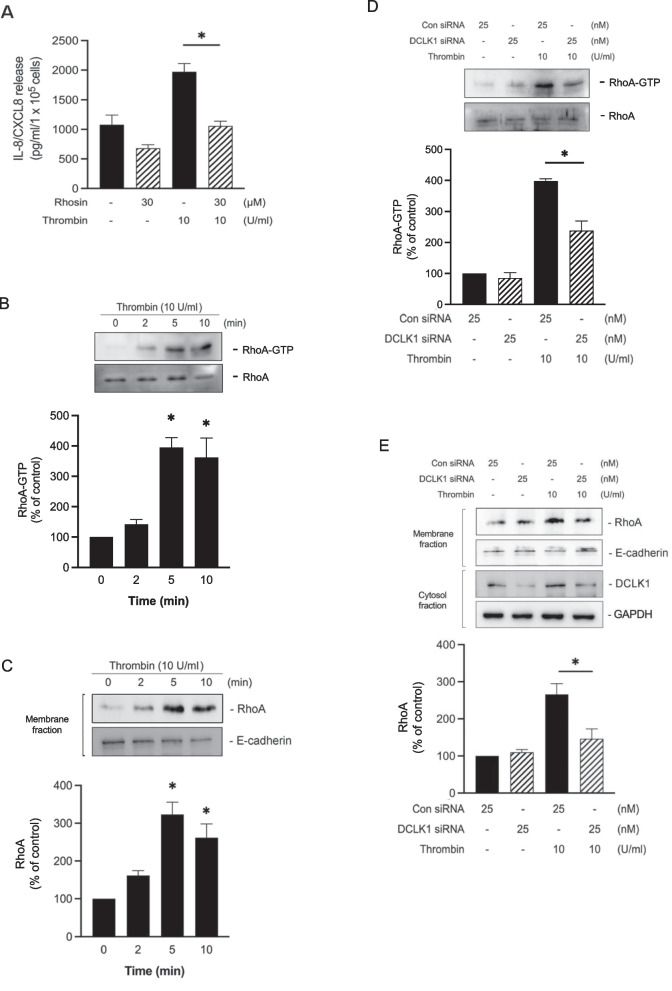
RhoA participates in thrombin-stimulated IL-8/CXCL8 expression in A549 cells. **A** A549 cells were treated with 30 μM Rhosin before being stimulated for the next 24 h with thrombin (10 U/ml). The level of IL-8/CXCL8 release was verified (n = 4, mean ± SEM, *p < 0.05 vs thrombin-stimulated cells). **B** A549 cells were treated with thrombin (10 U/ml) for the indicated periods. A Western blot pulldown assay was performed to determine RhoA activity (RhoA-GTP) level. Total RhoA level was used as a loading control (n = 4, mean ± SEM, *p < 0.05 vs non-stimulated cells). **C** Membrane fraction of A549 cells was extracted after thrombin (10 U/ml) for the indicated periods. Immunoblotting was performed to determine the protein level of RhoA in the membrane fraction. E-cadherin was used as a plasma membrane marker (n = 4, mean ± SEM, *p < 0.05 vs non-stimulated cells). **D** A549 cells were transfected with control (con siRNA) or DCLK1 siRNA for 48 h and subsequently treated with 10 U/ml thrombin for 5 min. A Western blot pulldown assay was performed to determine RhoA activity (RhoA-GTP) level. Total RhoA level was used as a loading control (n = 3, mean ± SEM, *p < 0.05 vs thrombin-stimulated cells). **E** A549 cells were transfected with control (con siRNA) or DCLK1 siRNA for 48 h and subsequently treated with 10 U/ml thrombin for 5 min. Membrane fraction were immunoblotted to determine the expression level of RhoA. (n = 4, mean ± SEM, *p < 0.05 vs thrombin-stimulated cells)

It was revealed that RhoA participates in the LPS-stimulated IL-8/CXCL8 production in respiratory epithelial cells [28]. Nevertheless, it is not known whether RhoA participates in thrombin-induced IL-8/CXCL8 release through DCLK1 in lung epithelial cells. To determine this, we used Rhosin (RhoA inhibitor). The findings suggested that the use of 30 μM Rhosin reduced IL-8/CXCL8 release stimulated by thrombin in A549 to 57.4 ± 8.9% (Fig. 5A). Next, we investigated whether thrombin can activate RhoA by performing a Rhotekin RBD pull-down assay for active RhoA since RhoA cycles between an inactive (GDP-bound) and an active form (GTP-bound). The time course of RhoA activity induced by thrombin is shown in Fig. 5B, which was characterized by an elevation in RhoA-GTP expression that peaked after 5 min of thrombin stimulation. RhoA is largely located in the cytosol but has been reported to translocate to the membrane when activated. Thus, changes in the amount of RhoA in the membrane have been used as an indicator of RhoA activation [40, 41, 42, 43]. We assessed RhoA expression level in the membrane fraction in A549 to evaluate RhoA activation. Thrombin was added to A549 in different time intervals. Thrombin induced the increase in the protein level of RhoA in the membrane fraction that peaked at 5 min in A549 (Fig. 5C). Next, we confirmed that DCLK siRNA (25 nM) attenuated thrombin-stimulated RhoA-GTP by 53.5 ± 11.1% (Fig. 5D) and membrane-associated RhoA stimulated by thrombin by 73.5 ± 22.9% in A549 cells (Fig. 5E). These findings imply that RhoA participates in thrombin-induced IL-8/CXCL8 expression via DCLK1.

### YAP is involved in thrombin-stimulated IL-8/CXCL8 expression

**Fig. 6:**
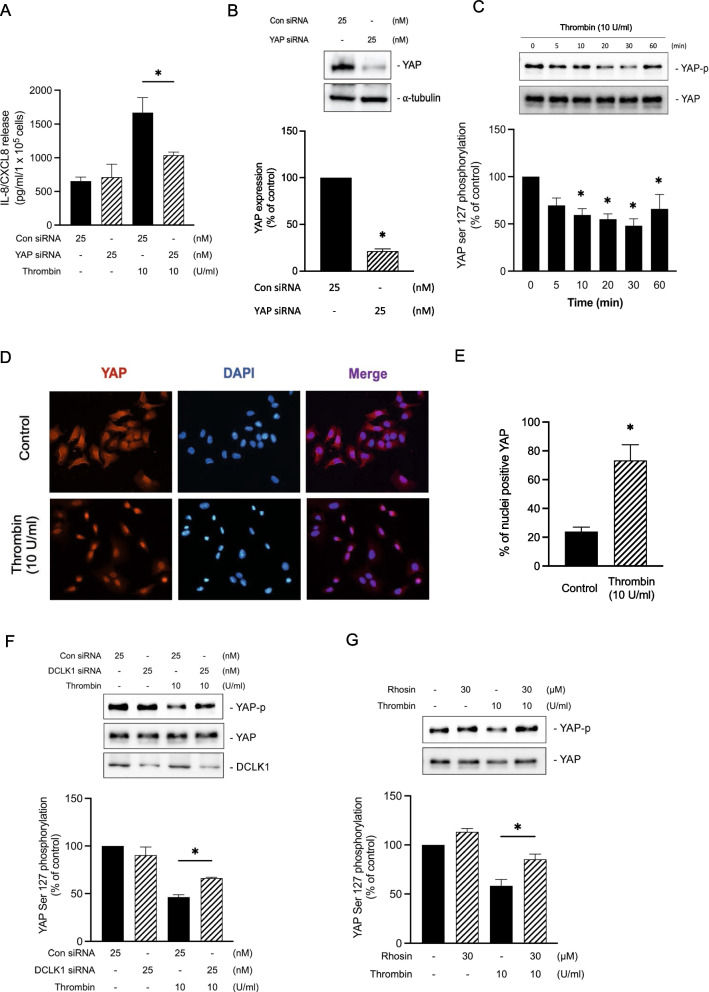
YAP participates in thrombin-stimulated IL-8/CXCL8 expression in A549 cells. **A** A549 cells were transfected with YAP siRNA or control (con siRNA) for 48 h before being treated for additional 24 h with thrombin (10 U/ml). ELISA was performed to measure the level of IL-8/CXCL8 release (n = 3, mean ± SEM, *p < 0.05 vs thrombin-stimulated group). **B** A549 cells were transfected with control (con siRNA) or YAP siRNA for 48 h (n = 3, mean ± SEM, *p < 0.05 vs control). Immunoblotting was performed to determine the expression level of YAP. **C.** A549 cells were stimulated by thrombin (10 U/ml) for 0–60 min. Immunoblotting was performed to determine the expression level of phospho-YAP Ser127 (n = 4, mean ± SEM, *p < 0.05 vs non-stimulated cells). **D** Representative immunofluorescence images showing YAP (red) nuclear localization after 30 min thrombin (10 U/ml) stimulation in A549 cells. Cell nuclei were stained with DAPI (blue) (n = 3, original magnification = ×20). **E** Quantitative analysis of the percent of cells with YAP nuclei positive staining without (control) or with thrombin stimulation in A594 cells (n = 3, mean ± SEM, *p < 0.05 vs control). **F** A549 cells were transfected with control (con siRNA) or DCLK1 siRNA for 48 h before being stimulated for another 24 h with thrombin (10 U/ml). Immunoblotting was performed to determine the expression level of phospho-YAP Ser127 (n = 3, mean ± SEM, *p < 0.05 vs thrombin-stimulated cells). **G** A549 cells were incubated with Rhosin for 30 min prior to treatment with thrombin for another 30 min. Immunoblotting was performed to determine the expression level of phospho-YAP Ser127 (n = 3, mean ± SEM, *p < 0.05 vs thrombin-stimulated cells)

YAP has been linked to COX-2 expression and the production of considerable PGE_2_, as well as in the contraction of bronchi [44]. In addition, RhoA-mediated thrombin induces YAP activation in human embryonic kidney cell line [30]. Using YAP siRNA (25 nM), we demonstrated that IL-8/CXCL8 release in response to thrombin stimulation was reduced by 67.9 ± 4.1% in A549 cells (Fig. 6A). In addition, the transfection of YAP siRNA functionally inhibited YAP protein expression in A549 cells by 78.6 ± 2.5% (Fig. 6B). Then, we explored whether thrombin could activate YAP. Previous studies have indicated that the dephosphorylation of YAP at Ser127 site promotes YAP activation and translocates from the cytosol into the nucleus, increasing target gene expression [30, 45]. Next, we observed whether thrombin would induce the dephosphorylation of YAP Ser127 and it showed that thrombin induced YAP activation via the dephosphorylation of YAP at the Ser127 position of A549, with a significant decrease within 30 min (Fig. 6C). Moreover, the immunofluorescence result showed that YAP translocated to the nucleus from the cytosol after thrombin stimulation in A549 (Fig. 6D), and the quantitative analysis of the images as shown in Fig. 6E suggested that the nuclei positive YAP in thrombin-stimulated cells was three times higher than in non-stimulated cells (control).

Next, we used DCLK1 siRNA and Rhosin to confirm whether thrombin induced YAP activation through DCLK1/RhoA pathway. The results showed that DCLK1 siRNA (25 nM) and Rhosin (30 μM) inhibited thrombin-induced YAP Ser127 dephosphorylation in A549 by 54.9 ± 11.7% and 41.0 ± 12.9% (Fig. 6F and Fig. 6G). The findings suggest that thrombin stimulates YAP activation through DCLK1/RhoA pathway.

### ERK participates in thrombin-induced DCLK1, RhoA, and YAP activation

**Fig. 7:**
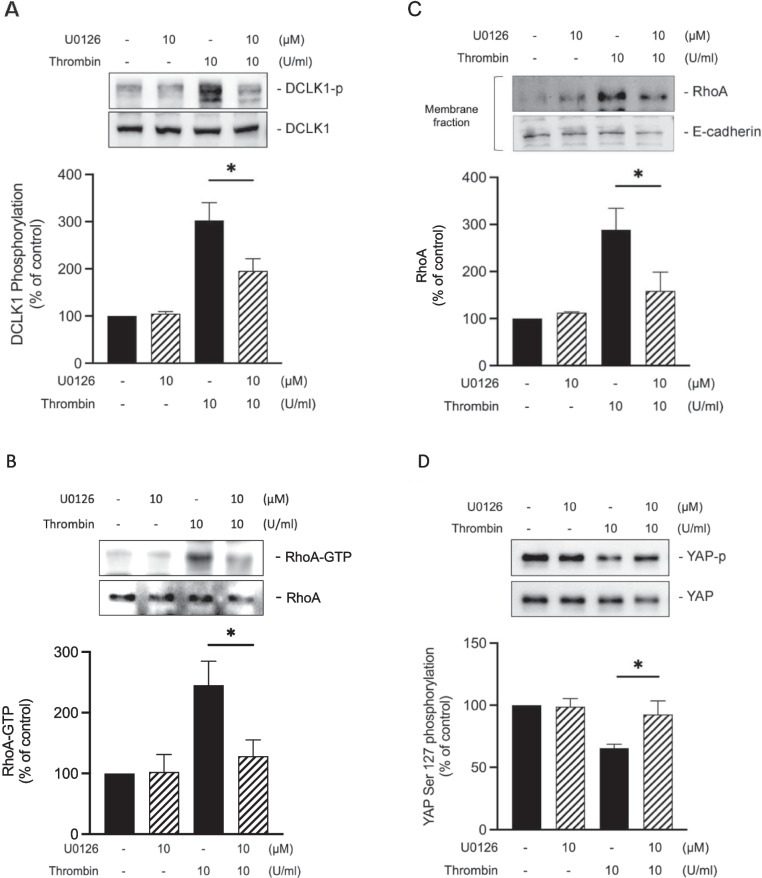
Thrombin induces DCLK1, RhoA, and YAP activation through the ERK pathway. **A** A549 cells were incubated with U0126 for 30 min prior to stimulation for another 20 min with thrombin (10 U/ml). Immunoblotting was performed to determine the expression level of phospho-DCLK1 Ser30 (n = 4, mean ± SEM, *p < 0.05 vs thrombin-stimulated cells). **B** A549 cells were incubated with U0126 (30 min) prior to thrombin (10 U/ml) treatment for another 5 min. A Western blot pulldown assay was performed to determine RhoA activity (RhoA-GTP) level. Total RhoA level was used as a loading control (n = 3, mean ± SEM, *p < 0.05 vs thrombin-stimulated cells). **C** A549 cells were incubated with U0126 for 30 min prior to thrombin (10 U/ml) treatment for another 5 min. Membrane fraction was immunoblotted to determine the expression level of RhoA (n = 3, mean ± SEM, *p < 0.05, vs thrombin-stimulated cells). **D** A549 cells were preincubated with U0126 for 30 min prior to treatment with thrombin for another 30 min. Immunoblotting was performed to determine the expression level of phospho-YAP Ser127 (n = 3, mean ± SEM, *p < 0.05, vs thrombin-stimulated cells)

Our previous study showed that ERK inhibitor (U0126) inhibits IL-8/CXCL8 release stimulated by thrombin in A549 cells, indicating ERK involvement in thrombin- induced IL-8/CXCL8 expression [46]. Moreover, DCLK1 phosphorylation is stimulated by ERK [20]. In this study, we confirmed that U0126 inhibited thrombin-induced DCLK1 Ser30 phosphorylation (Fig. 7A), RhoA activation (Fig. 7B and Fig. 7C) and YAP dephosphorylation by 55.0 ± 12.7%, 82.0 ± 18.5%, 75.3 ± 21.1% and 68.5 ± 25.8%, respectively (Fig. 7D). These findings indicate that DCLK1/RhoA/YAP pathway is implicated in IL-8/CXCL8 expression stimulated by thrombin, and that its activation is mediated by ERK signaling.

### DCLK1 participates in thrombin-induced YAP-p65 interaction to promote IL-8/CXCL8 expression

**Fig. 8:**
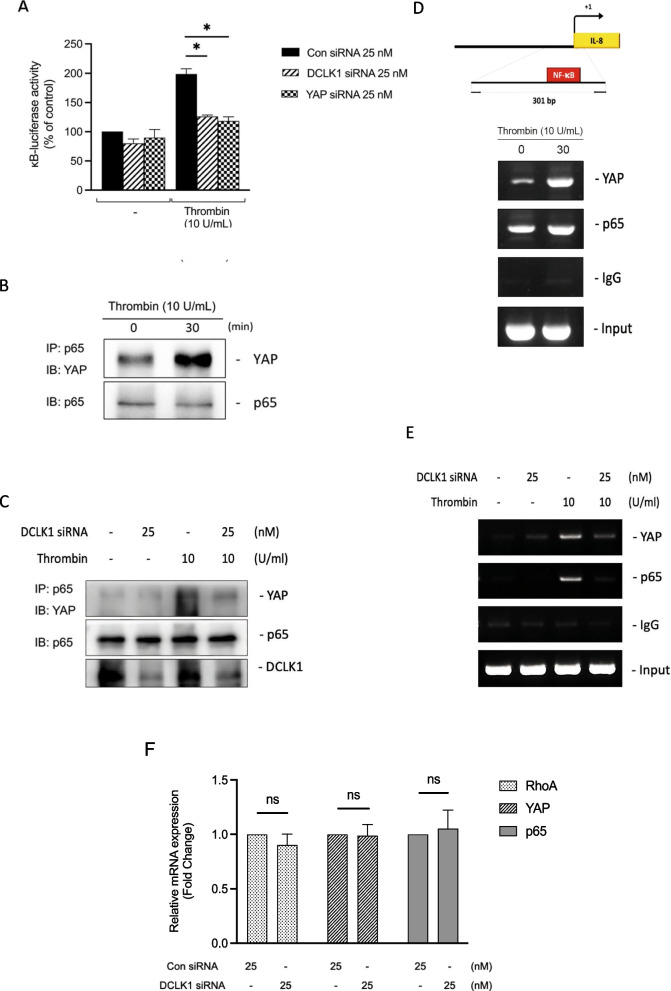
Thrombin stimulates YAP and p65 formation in A549 cells. **A** A549 cells were transfected with pGL2-ELAM-κB-Luc (0.5 μg) and pBK-CMV-lacZ (0.5 μg), control (con siRNA), or DCLK1 siRNA, or YAP siRNA for 48 h before being incubated for 16 h with thrombin (10 U/ml). Cells were harvested for a luciferase activity assay (n = 3, mean ± SEM, *p < 0.05 vs thrombin-treated cells). **B** A549 cells were collected and immunoprecipitated with anti-NF-κB p65 antibody after being stimulated for 30 min with thrombin (10 U/ml). Immunoblotting was performed to detect the immunoprecipitated complex with anti-YAP and anti-NF-κB p65 antibodies. The y7immunoprecipitated complex was identified using immunoblotting with anti-YAP and anti-NF-κB p65 antibodies (n = 3). **C** A549 cells were transfected with control (con siRNA) or DCLK1 siRNA for 48 h and subsequently collected and immunoprecipitated with anti-NF-κB p65 antibody after being stimulated for 30 min with thrombin (10 U/ml). Immunoblotting was performed to detect the immunoprecipitated complex with anti-YAP and anti-NF-κB p65 antibodies. The immunoprecipitated complex was identified using immunoblotting with anti-YAP and anti-NF-κB p65 antibodies (n = 3). **D** A549 cells were treated with 10 U/m thrombin for 30 min. Cell lysates were used for ChIP analysis with antibodies against YAP or p65. PCR was used to detect YAP or p65 binding on IL-8/CXCL8 promoter. Input, a positive control. IgG, a negative control (n = 4). **E** A549 cells were transfected with control (con siRNA) or DCLK1 siRNA for 48 h and subsequently the cell lysates were used for ChIP analysis with antibodies against YAP or p65. PCR was used to detect YAP or p65 binding on IL-8/CXCL8 promoter. Input, a positive control. IgG, a negative control (n = 3). **F** RhoA, YAP, and p65 mRNA expression levels after transfection of A549 cells with control (con siRNA) or DCLK1 siRNA (n = 3–4, mean ± SEM, *p < 0.05 vs control)

Thrombin has previously been shown to activate IκB kinase αβ (IKKαβ), which leads to the translocation of p65 to the nucleus from the cytosol and eventually induces the expression of IL-8/CXCL8 [9]. Next, we investigated whether NF-κB activation occurs via the DCLK1 and YAP pathways. We used the κB-luciferase reporter gene to evaluate NF-κB activation. After transfecting the A549 with DCLK1 siRNA, YAP siRNA, and κB-luciferase reporter plasmids, thrombin stimulation was added to observe the inhibition of κB-luciferase activity by DCLK1 siRNA and YAP siRNA. The result showed that DCLK1 siRNA and YAP siRNA inhibited κB-luciferase activity in A549 by 53.4 ± 2.4% and 71.1 ± 7.1%, respectively (Fig. 8A). Furthermore, we performed immunoprecipitation to investigate whether the transcription co-activator YAP will bind to p65 as a transcription factor to induce target gene expression. As shown in Fig. 8B, YAP interacted with p65 after 30 min thrombin stimulation. Furthermore, we confirmed that DCLK1 siRNA inhibited thrombin-induced YAP and p65 interaction, showing that DCLK1 is involved in YAP and p65 interaction stimulated by thrombin in A549 (Fig. 8C). Next, we used ChIP to explore YAP and p65 binding to IL-8/CXCL8 promoter. We proved that thrombin stimulated YAP and p65 to bind to the promoter of IL-8/CXCL8 (Fig. 8D). Next, we used DCLK1 siRNA to confirm whether DCLK1 is involved in thrombin-induced YAP and p65 binding to IL-8/CXCL8 promoter. Our findings suggested that DCLK1 siRNA suppressed YAP and p65 binding to IL-8/CXCL8 promoter induced by thrombin in A549 cells (Fig. 8E). We then observed whether DCLK1 siRNA also inhibits the expression of RhoA, YAP, and p65 in gene expression level. We found that DCLK1 siRNA could not suppress the mRNA expression level of RhoA, YAP, and p65 in A549 cells (Fig. 8F). These findings showed that DCLK1 and YAP activation is required for thrombin-stimulated NF-κB activation by the formation of YAP/p65 complex that binds to IL-8/CXCL8 promoter in A549.

## Discussion

Our findings revealed an important role of DCLK1, a marker of some cancer stem cells, in mediating thrombin-stimulated IL-8/CXCL8 expression in human lung epithelial cells. As IL-8/CXCL8 is a potent chemoattractant of neutrophils, its increase will be followed by elevated recruitment of neutrophils to the inflamed area, exacerbating the inflammatory response that leads to severe asthma [47, 48]. Asthma is linked with increased thrombin generation and fibrinolysis impairment [49]. A large amount of thrombin was observed in the asthma patients’ sputum and bronchoalveolar and plays a vital role in the pathological process [10]. DCLK1 is highly expressed in several types of inflammation-associated cancer, such as pancreas, colon, liver, esophageal, and lung cancer [50, 51]. The activation of S100A9/NF-κB pathway in hepatitis C virus through DCLK1 induced cirrhosis, and DCLK1 overexpression was positively correlated with the expression of inflammatory genes [23]. These findings support a possible association between DCLK1 and asthma as an inflammatory disease. This study demonstrates that IL-8/CXCL8, thrombin, and DCLK1 are overexpressed in the bronchial tissues of severe asthma patients and OVA-induced asthmatic mice. Thrombin activates ERK, DCLK1, and RhoA, resulting in YAP activation and translocation to the nucleus from the cytosol, where it interacts with NF-κB p65 and binds to IL-8/CXCL8 promoter to induce its production in lung epithelial cells.

We previously demonstrated that ERK participates in thrombin-stimulated IL-8/CXCL8 production via MEKK1, ERK, and RSK1 activation [46]. Another study suggested that ERK induces DCLK1 Ser30 phosphorylation, which can increase the expression of endocrine melanocyte pro-opiomelanocortin [20]. We recently found that U0126 (ERK inhibitor) inhibits DCLK1 Ser30 phosphorylation in A549 cells, showing that ERK is participated in DCLK1 activation by thrombin stimulation. DCLK1 siRNA and LRRK2-IN-1 (DCLK1 inhibitor) ameliorated thrombin-induced IL-8/CXCL8 release in BEAS-2B and A549 cells. We used DCLK1 siRNA to specifically target DCLK1 and found that it inhibits thrombin-induced IL-8/CXCL8 release without affecting basal level in A549 cells. In this study, we found that LRRK2-IN-1, a small molecule kinase inhibitor, sometimes inhibits basal level of IL-8/CXCL8 release. It will be due to LRRK2-IN-1 that can yield off-target effects by inducing changes in molecules other than the one specifically targeted or by indirect “pathway cross-talk” effects [52]. Previous study also revealed a significant off-target effects of LRRK2-IN-1 [53]. Our findings show that DCLK1 is a downstream molecule involved in ERK-mediated IL-8/CXCL8 expression after thrombin stimulation in lung epithelial cells.

DCLK1 regulates PD-L1 expression in pancreatic tumor via the Hippo pathway [54]. The Hippo pathway was discovered to be critical in controlling cell growth and organ size, but recent researches have revealed that it also participates in tumorigenesis [55, 56, 57]. YAP is a major downstream molecule and transcriptional coactivator of Hippo signaling, and it is involved in cancer and asthma pathogenesis [31, 32]. Previous study has indicated that a large amount of YAP can be used as a biomarker to assess asthma severity [33]. In addition, RhoA can mediate YAP activation. When RhoA is activated, it inhibits LATS1/2 phosphorylation by the Hippo kinase via F-actin and promotes YAP dephosphorylation of its Ser127, resulting in YAP activation and translocation into the nucleus. As YAP lacks a DNA binding site, it must be combined with transcription factors, such as TEAD or p65, to increase gene transcription [34, 45]. CD44, a transmembrane glycoprotein, regulates YAP expression via RhoA in mediating cell apoptosis, migration, and proliferation in A549 and HepG2 cells [58]. Furthermore, RhoA is required for lysophosphatidic acid (LPA)-induced YAP dephosphorylation in ovarian cancer cells for long-term migration [59]. In addition, thrombin can increase the activation of RhoA’s active mediator YAP via PAR1 [30]. Moreover, DCLK1 silencing by microRNA-195 can downregulate RhoA expression in pancreatic cancer cells, indicating that DCLK1 also has an interaction with RhoA [60]. However, it's unclear whether RhoA and YAP are implicated in DCLK1-mediated thrombin-induced IL-8/CXCL8 expression. Here, we identified that the use of Rhosin and siRNA YAP inhibits thrombin-stimulated IL-8/CXCL8 expression in A549 cells. Moreover, thrombin-induced RhoA activation and YAP Ser127 dephosphorylation can be inhibited by DCLK1 siRNA. Thrombin-stimulated YAP dephosphorylation is also inhibited by Rhosin, indicating that YAP activation is regulated through RhoA activation.

Previous research has revealed that DCLK1 regulates p65 as a prosurvival signaling that is essential for cancer formation and progression [22]. It has been suggested that thrombin induces NF-κB p65 translocation to the nucleus from the cytosol and regulates IL-8/CXCL8 production in lung epithelial cells through ERK activation [9, 11]. Another study also supported the involvement of DCLK1 in activating NF-κB pathway to induce cirrhosis in hepatitis C virus [23]. In addition, we previously suggested that thrombin stimulates the expression of IL-8/CXCL8 in human lung epithelial cells by activating NF-κB via c-Src [9]. RhoA is involved in LPS-induced respiratory tract epithelial cell NF-κB activation and IL-8/CXCL8 expression [28]. Another investigation also showed the involvement of YAP and p65 NF-κB in increasing inflammation and breast cancer cell migration after TNF-⍺ stimulation [45]. However, it remains unclear whether DCLK1 mediates thrombin-stimulated IL-8/CXCL8 release through NF-κB activation. In this study, we found that thrombin stimulates the recruitment of YAP and the p65 complex to the NF-κB-binding region of the IL-8/CXCL8 promoter, which is inhibited by DCLK1 siRNA. These results demonstrate that YAP interacts with p65 and binds to the IL-8/CXCL8 promoter at the κB-binding site, hence increasing IL-8/CXCL8 expression through DCLK1.

We found that the inhibition of DCLK1 cannot completely attenuate thrombin-induced IL-8/CXCL8 release through RhoA/YAP/NF-κB signaling. This is attributable to the various transcription factors that regulate IL-8/CXCL8 expression. Taken together, ERK, DCLK1, RhoA, YAP, and NF-κB signaling pathways participates in thrombin-stimulated IL-8/CXCL8 expression in lung epithelial cells.

Although our findings strongly imply that DCLK1 is essential in mediating thrombin-stimulated IL-8/CXCL8 expression, it is still unknown how DCLK1 expression is regulated in lung epithelial cells. Prior study has shown that severe asthma patients exhibit increased IL-17 levels in their airways [24]. Moreover, IL-17 drives the upregulation of DCLK1 expression in a bleomycin-induced lung fibrosis mice model and pancreatic intraepithelial neoplasia cells [25, 26]. However, the regulators of DCLK1 expression in lung epithelial cells in severe asthma remain unclear, and further studies are required to address these issues. Furthermore, while we focus on lung epithelial cells in this study, other cells in the lungs also play a role in chronic airway inflammation. As previous findings showed that fibrocytes, fibroblasts, and smooth muscle cells contributes in the pathogenesis airway fibrosis in chronic obstructive asthma patients [61, 62, 63], further studies to explore the involvement of DCLK1 in those cells also need to be performed.

The clinical and experimental study indicates that high level of IL-8/CXCL8, thrombin, and DCLK1 is related with asthma severity. This investigation revealed that DCLK1 participates in the increase of IL-8/CXCL8 expression stimulated by thrombin in lung epithelial cells. As IL-8/CXCL8 is a potent chemoattractant of neutrophil and airway neutrophilia is correlated with asthma severity, DCLK1 involvement in the thrombin-stimulated IL-8/CXCL8 expression pathway indicates that DCLK1 might serve as a new biological marker of severe asthma. As the therapy for severe asthma remains challenging, the finding of DCLK1 as a potential marker may be beneficial as a target therapy.

## Conclusions

**Fig. 9:**
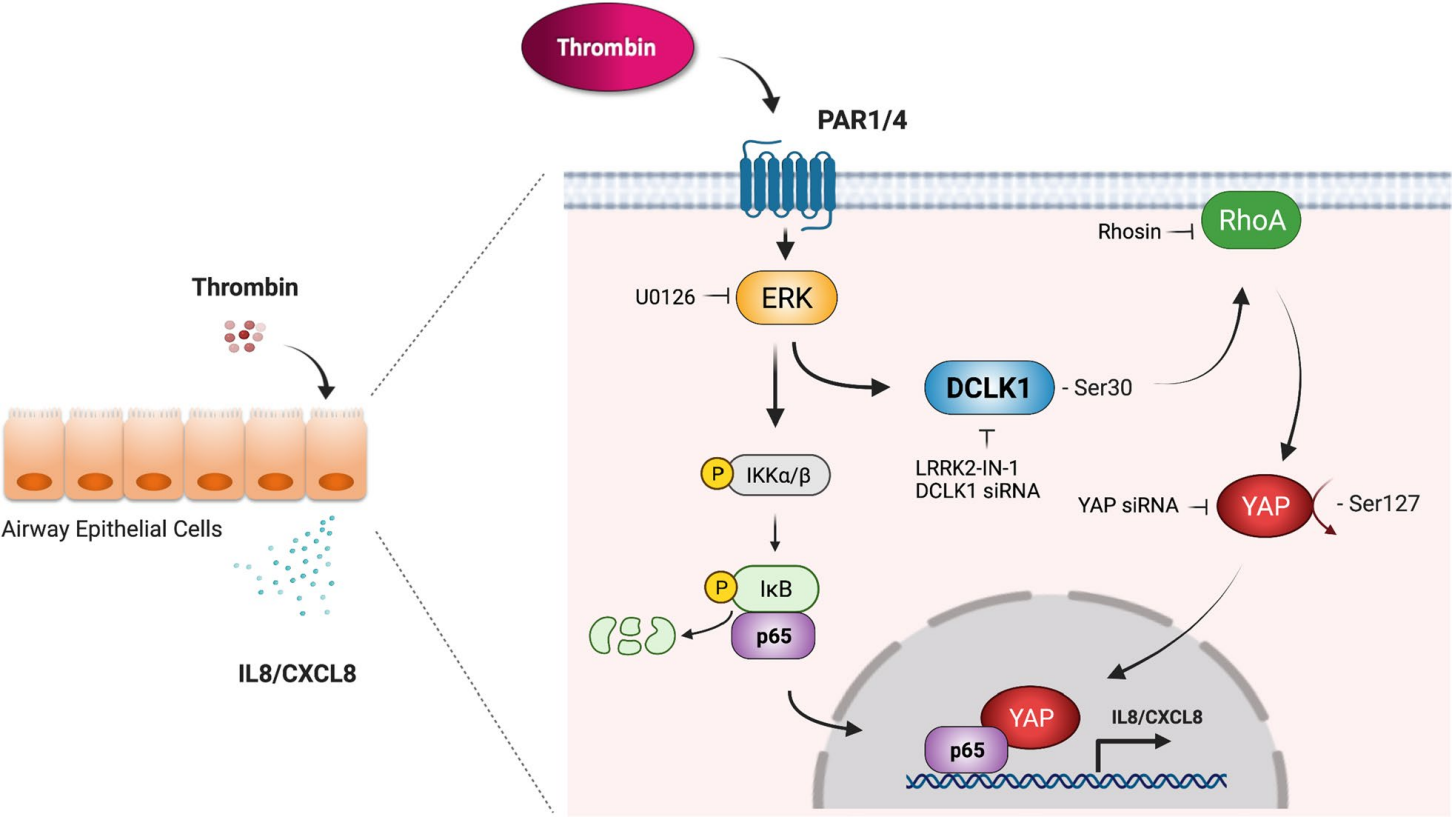
Schematic summary of thrombin-induced IL-8/CXCL8 expression signaling pathway involving DCLK1 in lung epithelial cells. It has previously been revealed that thrombin-induced ERK activation increases IKKα/β phosphorylation, which promotes p65 to translocate to the nucleus from the cytosol, resulting in IL-8/CXCL8 expression. In this report, DCLK1 is involved in thrombin-induced IL-8/CXCL8 expression via ERK and then mediates the activation of RhoA and YAP. Once YAP is activated, it moves to nucleus from cytosol, where it binds to the transcription factor p65, increasing IL-8/CXCL8 expression in human lung epithelial cells. This image was created with BioRender (https://biorender.com/)

This report demonstrates that DCLK1 is upregulated in lung tissue in severe asthma and proves the role of DCLK1 in mediating thrombin-induced IL-8/CXCL8 expression in human lung epithelial cells. The findings suggest that DCLK1 mediates IL-8/CXCL8 expression in response to thrombin stimulation via RhoA and YAP signaling pathways in human lung epithelial cells. Figure 9 illustrates the signaling pathways of thrombin-induced IL-8/CXCL8 expression in human lung epithelial cells. Our findings indicate the abundant expression of DCLK1 in lung tissue of severe asthma patients and plays a critical role in increasing the expression of IL-8/CXCL8 after thrombin stimulation in lung epithelial cells, which can explain the molecular mechanism of severe asthma and identify potential drug targets.

## Data Availability

The datasets used and/or analysed during the current study are available from the corresponding author on reasonable request.

## Abbreviations

BEGM: Bronchial epithelial growth medium
BSA: Bovine serum albumin
ChIP: Chromatin immunoprecipitation
DMEM/F-12: Dulbecco’s modified Eagle’s medium/Nutrient Mixture F-12
DCLK-1: Doublecortin-like kinase-1
ECL: Enhanced chemiluminescence
ERK: Extracellular signal‐regulated kinase
FBS: Fetal bovine serum
HRP: Horseradish peroxidase
IHC: Immunohistochemistry
IF: Immunofluorescence
IP: Immunoprecipitation
IL-8/CXCL8: Interleukin 8/C-X-C motif chemokine ligand 8
MIP-2: Macrophage inflammatory protein 2
NF-κB: Nuclear factor kappa B
OVA: Ovalbumin
PBS: Phosphate-buffered saline
PVDF: Polyvinylidene difluoride
RhoA: Ras homolog family member A
SDS-PAGE: Sodium dodecyl sulfate polyacrylamide gel electrophoresis
siRNA: Small interfering RNA
YAP: Yes-associated protein
